# Development of an eco-friendly HPLC method for the stability indicating assay of binary mixture of ibuprofen and phenylephrine

**DOI:** 10.1186/s13065-023-01056-4

**Published:** 2023-10-24

**Authors:** Khadiga M. Kelani, Yasmin M. Fayez, Ahmed M. Abdel-Raoof, Reham A. Fekry, Said A. Hassan

**Affiliations:** 1 Analytical Chemistry Department, Faculty of Pharmacy, Cairo University, Kasr El-Aini Street, Cairo, 11562 Egypt; 2 Analytical Chemistry Department, Faculty of Pharmacy (Boys), AL-Azhar University, Nasr City, Cairo, 11751 Egypt; 3 Analytical Chemistry Department, Faculty of Pharmacy, Modern University for Technology and Information, El-hadaba El-Wosta, Mokatam, 5th District, Cairo, Egypt; 4 Analytical Chemistry Department, Faculty of Pharmacy, Misr University for Science and Technology, Al-Motamayez District, P.O. Box 77, 6th of October City, Egypt

**Keywords:** Degradation, Green analytical chemistry, HPLC, Ibuprofen, Phenylephrine, Stability indicating assay, Molecular dynamic simulation

## Abstract

The development and validation of the stability indicating HPLC technique has contributed to the understanding of the stability profile of ibuprofen (IBU) and phenylephrine (PHE). Stability profile was achieved for PHE; the drug was found to be liable to be influenced by stress oxidative conditions; two oxidative degradants (Deg1 & Deg2) were formed and their structures were confirmed using IR and mass spectrometry. The drugs and degradation products were successfully separated using a gradient elution method on YMC-C8 column with 0.1% hexanesulfonic acid and acetonitrile as a mobile phase at pH 6.6. The flow rate was 1.0 mL/min, and a diode array detector operating at 220 nm was used for UV detection. The retention times of degradants Deg1, Deg2, ibuprofen (IBU), and phenylephrine hydrochloride (PHE) were 2.0, 2.2, 3.2 and 7.0 min, respectively. The proposed method was validated with respect to linearity, accuracy, precision, specificity, and robustness using ICH guidelines. The linearities of ibuprofen and phenylephrine hydrochloride were in the range of 10–100 μg/mL and 0.3–10 μg/mL, respectively. The % recoveries of the two drugs were found to be 100.75 ± 1.44%, 99.67% ± 1.67, and the LOD was found to be 2.75/mL and 0.09/mL for IBU, and PHE, respectively. The method was successfully applied to the estimation of ibuprofen and phenylephrine hydrochloride combination in pharmaceutical dosage form. The proposed technique was validated using ICH guidelines and its greenness was assessed according to Analytical Eco Scale metric (AES). Molecular docking was used to assess the two drugs and PHE oxidative degradants interaction with the stationary phase and to confirm the outcomes of the proposed method with regard to the order of elution of the two drugs and PHE degradation products. Eco-friendly and environmental safety were assessed through the application of one of the most applicable greenness assessment tool; Analytical Eco Scale metric (AES).

## Introduction

**Fig. 1:**
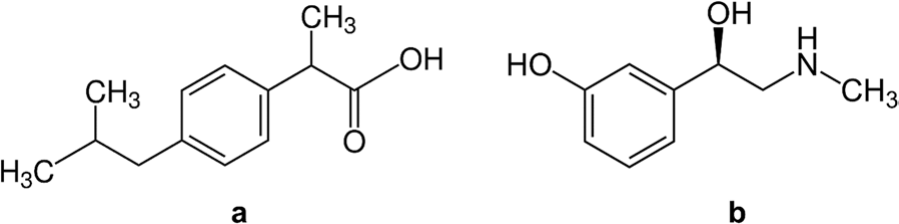
Chemical structures of IBU (**a**) and PHE (**b**)

Ibuprofen (IBU), (2RS)-2-[4-(2-Methylpropyl)phenyl]propanoic acid [1] (Fig. 1a) is used to relieve pain, fever and inflammation as it is a nonsteroidal anti-inflammatory medicine. It acts mainly as an anti-inflammatory, antipyretic and analgesic drug through inhibiting the cyclooxygenase-2 enzyme [2]. Phenylephrine (PHE), (1R)-1-(3-Hydroxyphenyl)-2-(methylamino)ethanol [1] (Fig. 1b) is a medicine mainly utilized as a decongestant, to enlarge the pupil, to raise the blood pressure, and to treat hemorrhoids [3]. The combination dosage form of IBU and PHE is indicated to treat the symptoms of the common cold and flu [4], such as nasal congestion, headache, fever and moderate aches and pains. It is available in the market in the form of coated tablets under trade name Grippostad®.

Stability studies can help to identify the probable degradation pathways under various stress conditions [5]. They are absolutely necessary for shelf life calculation of pharmaceutical products and confirmation of quality, safety and efficiency [6]. Different techniques of analytical chemistry have been used for development of stability-indicating assay methods such as spectrophotometry [7, 8] and electrochemistry [9, 10] with HPLC representing the main technique of choice for this application [11–14].

Literature survey revealed that few techniques are available for quantification of PHE and IBU simultaneously in their binary combination such as spectrophotometry [15], spectrofluorimetry [16] and HPLC [17, 18]. The two drugs were subjected to stability studies in their mixtures with other drugs [19–25], but no method was developed for the stability indicating assay of this binary mixture. IBU was susceptible only to strong oxidative and thermal degradation [23–25]. PHE was reported to be more stable toward acidic, alkaline, and thermal conditions [19–22]; it is more susceptible to oxidative conditions [20].

Health risks associated with PHE degradation products were reported [26–31]. Therefore, degradation of PHE is a major concern in pharmaceutical formulation’s development in comparison to IBU, and the focus of this study was to investigate PHE degradation.

Green analytical chemistry (GAC) is a growing field which is connected with developing analytical methods that eliminate or minimize the harmful effects of various organic solvents on health as well as on the environment [32]. Nowadays, protection of the environment and personal health are carefully considered in the field of chemistry, that led to establishing guidelines about how to work according to green principles and increased the number of articles discussing GAC [33, 34]. It has been considered in different techniques such as spectrophotometry [35], spectrofluorimetry [36, 37], electrochemistry [38] and chromatography [39, 40].

Chromatographic techniques are the most common in the pharmaceutical analysis with high-performance liquid chromatography in the reverse-phase separation mode (RP-HPLC), accounts for more than 90% of separations in modern analytical laboratories [41]. However, they suffer from being not full green techniques because of pretreatment steps, long analysis times, too many trials for method optimization, high expensive instruments, high energy consumption, large waste and limited solvents options [42]. For reduction of chemical hazards released into the environment, planning for the greenness of analytical methods should therefore be assured before practical trials in a laboratory. Moreover, inclusion of the evaluation of greenness of analytical methods in method validation protocols is strongly recommended. One of the main challenges is the evaluation of the greenness of these techniques [42].

Several methods for evaluating greenness of analytical methods were developed [40]. Examples of these methods are the National Environmental Methods Index (NEMI) [43], Analytical Eco Scale (AES) [44], the Green Analytical Procedure Index (GAPI) [45] and Analytical Greenness metric (AGREE) [46]. Analytical Eco Scale (AES) was reported to provide reliable and precise results about method greenness [34, 40–53].

The molecular dynamic simulation technique (MDS) is an important computational tool for understanding the system's dynamic development, determining the physical foundation of the structure, assessing the stability of interactions at the molecular level, and verifying practical work outcomes. As a result, the primary goal of using MDS, which was employed for the first time in chromatographic approaches, was to evaluate the introduced drug's interaction with the stationary phase and to confirm the results of the proposed method by revealing which drug will be less retained in the stationary phase and which one will be more retained [54].

The aim of this work was to develop an eco-friendly HPLC method for stability indicating assay of the binary mixture of IBU and PHE. Due to the significance of PHE degradation on health, its degradation products were prepared and characterized. MDS was used to analyze the elution behavior of the intact drugs and degradants and describe their interaction with the stationary phase. AES metric was used for calculating of greenness of this method in comparison with a published method.

## Experimental

### Instrumentation

HPLC system (Agilent 1100 Model G1315A) with a photodiode array detector (DAD) and 100 µL injection loop and auto sampler. TLC plates (10 × 20 cm, 0.25 mm thickness) pre-coated with silica gel GF254 (Merck, Darmstadt, Germany).

### Materials and reagents

#### Pure standards

IBU and PHE were kindly supplied by Global Napi Pharmaceuticals (Egypt); their purities were found to be 99.62 ± 1.11% and 100.02 ± 1.14%, respectively, in accordance to the BP methods [1].

#### Pharmaceutical dosage form

Grippostad® film coated tablets produced by ES, Laboratorio Stada, Spain. Each tablet contains 200 mg of ibuprofen and 5 mg of phenylephrine.

#### Chemicals and reagents

HPLC grade acetonitrile (Merck, Darmstadt), hexansulfonic acid sodium salt monohydrate (Merck, Darmstadt), methanol (Sigma-Aldrich, Germany), ethyl acetate (Adwic-Egypt) and 30% ammonia solution (Adwic-Egypt).

#### Solutions

- Standard stock solutions of 1.0 mg/mL of IBU and PHE were prepared using methanol.
- Working standard solutions were prepared by diluting 10 mL of the stock solution of IBU and PHE with the mobile phase in100-mL volumetric flasks to get solution of (100 µg /mL).
- Laboratory-prepared mixtures containing various concentrations of IBU (10–100 µg/mL) and PHE (0.3–10 µg/mL) and different ratios of PHE degradants (10–90%) were prepared by transferring appropriate accurately measured volumes of their stock solutions into 10-mL volumetric flasks and completing to volume with the HPLC mobile phase.

### Chromatographic conditions

Gradient separation was carried out at 35 °C on YMC-C8 column (100 × 4.6 mm, 3 µm) using a mobile phase consisting of 0.1% hexanesulfonic acid and acetonitrile. The gradient started with 0.1% hexanesulfonic acid and acetonitrile at ratio 80:20 v/v; after 3 min the ratio was changed to 60:40 v/v till the end of the run (7 min). The mobile phase was de-gassed and pumped at a flow rate of 1 mL/min. IBU, PHE and PHE degradants solutions were injected in triplicates (20 µL) and detection was achieved at 220 nm.

### Procedures

#### Preparation of PHE degradants

Oxidative degradation was performed by refluxing 100 mg of PHE powder with 25 mL of 3% H_2_O_2_ for 3 h under dark conditions. Excess H_2_O_2_ was then removed by evaporation. The solution was cooled, transferred into 25-mL volumetric flask and the volume completed to mark with methanol. Complete degradation of the drug was confirmed by TLC. Silica gel 60 F_254_ plates were used to check the disappearance of intact drug spot (at R_f_ 0.12) and appearance of two spots of degradation products (at R_f_ 0.45 and 0.55) using ethyl acetate–methanol-30% ammonia solution (8:2:0.1 v/v) as the developing mobile phase. Preparative TLC was used for the separation of drug and its degradation products. Each of the separated spots was scratched and extracted twice with methanol. Each of the resulting solutions was dried at 100 °C to obtain pure solid form for further analysis.

#### Construction of the calibration curves

Accurately measured aliquots equivalent to 100–1000 µg of IBU and 3–100 µg of PHE were precisely transferred from the standard working solutions (200 µg/mL) into two separate sets of 10-mL volumetric flasks and the volumes were completed using the mobile phase. Membrane filter (0.45 µm) was used to filter the resulting solutions and 20 µL aliquots of the resulting solutions were injected in triplicates into the column under the above described chromatographic conditions. Calibration curves were constructed by plotting the integrated peak areas against the corresponding concentrations (in μg).

#### Assay of laboratory prepared mixtures by the proposed method

Various combinations of IBU, PHE and PHE degradants were prepared and mixed in various proportions as outlined in the solutions section. The prepared solutions were then analyzed by the developed method as described under construction of calibration curve.

#### Application of the proposed method to tablet dosage form

Ten of Grippostad® film coated tablets (labelled to contain 200 mg of IBU and 5 mg PHE per tablet) were precisely weighed, the tablets were first stripped of the film before being finely powdered. A precisely weighed portion containing 200 mg of IBU and 5 mg of PHE was sonicated in 30 mL methanol for 10 min before being filtered into a 100-mL volumetric flask. The residues were rinsed multiple times with 10 mL of methanol, and the solutions were adjusted to the mark with the same solvent. A suitable aliquot was then diluted with the mobile phase in order to obtain a solution containing 40 and 1 µg /mL of IBU and PHE, respectively.

### Molecular dynamic simulation

Molecular dynamic simulation was used to assess drugs interaction with the stationary phase and to reveal which drug will be more and which one will be less retained in the stationary phase.

### Assessment of the greenness of the developed method

Assessment of the proposed method greenness was carried out using the Analytical Eco Scale (AES) tool [44].

## Results and discussion

Health hazards associated with the degradation of PHE is well documented as mentioned in the introduction, so its degradation was the core of our stability study. The possible degradation pathways and products of PHE and IBU were studied at similar stress conditions for developing eco-friendly HPLC stability indicating method for assay of the binary mixture of PHE and IBU. PHE was reported to be more stable toward acidic, alkaline, and thermal conditions [19–22] but more susceptible to oxidative conditions producing two degradation products [20]. IBU was reported to be susceptible only to strong oxidative and thermal degradation [23–25].

Although the toxicological effects of IBU and PHE degradation products remain largely unknown, a recent study highlighted that some of the degradation products of ibuprofen can be more toxic to human kidney cell and liver cell lines [31]. PHE photodegradation may produce epinephrine [26], which has stronger agonist effect on adrenergic receptors [27]. Acetylated degradation products of PHE in tablets containing aspirin were previously detected and characterized [28], and a possible decomposition pathway for PHE may form parkinsonism‐inducing agents [29, 30]. Therefore, degradation of PHE is a major concern in pharmaceutical formulation’s development in comparison to IBU. Chemical degradation of drugs may also result in loss of drug potency; clinical use of a medicine is unacceptable in case of relatively high degradation. In addition, when a drug dosage form is altered, the stability of the drug may be affected [55].

### Preparation and structure elucidation of PHE degradation products

**Fig. 2:**
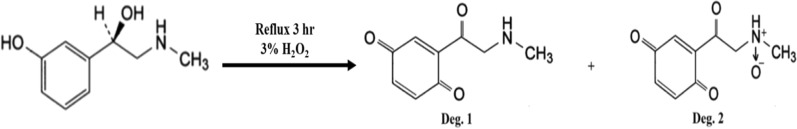
Suggested oxidative degradation pathway for PHE with two degradation products (Deg1 & Deg2)

**Fig. 3:**
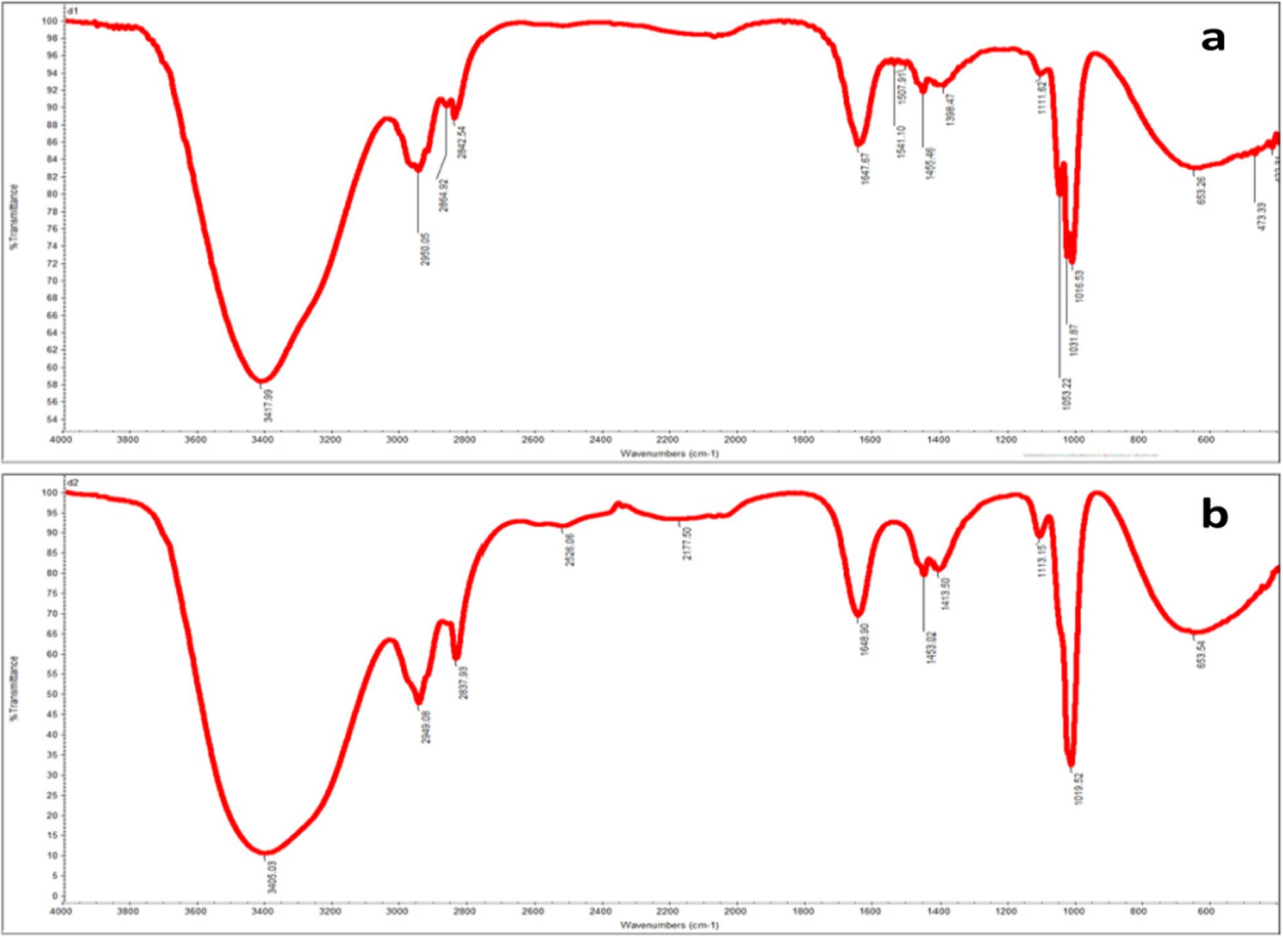
IR Spectrum of PHE degradants: (**a**) Deg1 and (**b**) Deg2

**Fig. 4:**
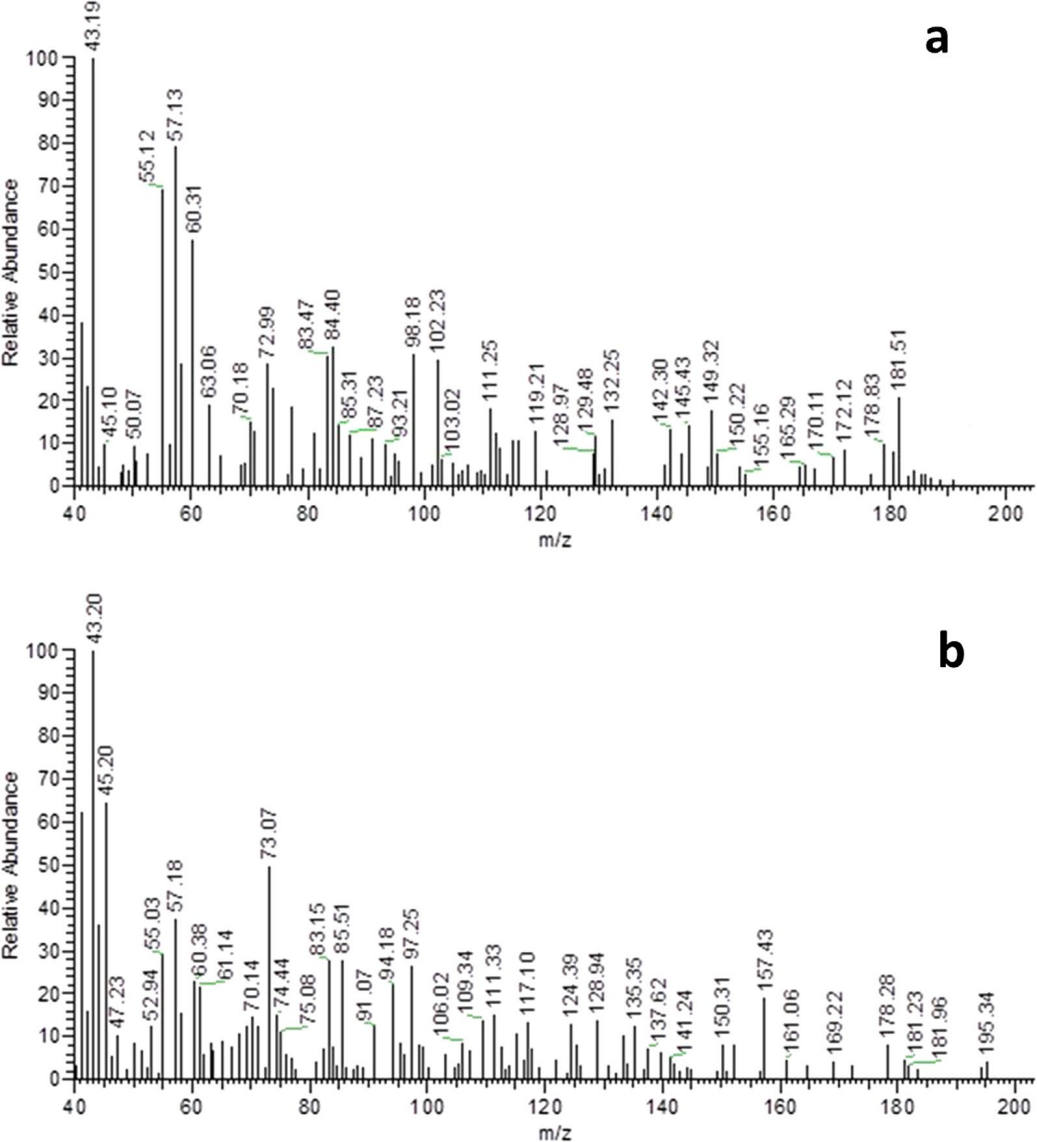
Mass spectra of PHE degradants: (**a**) Deg1 and (**b**) Deg2

Development of stability-indicating analytical method requires the study of the possible degradation products and pathways for drugs at similar stress conditions. In our work, IBU and PHE were subjected to the same oxidative stress conditions (refluxing with 3% H_2_O_2_ for 3 h) and their degradation was monitored using TLC. Silica gel 60 F_254_ plates were used using ethyl acetate–methanol-30% ammonia solution (8:2:0.1 v/v) as the developing mobile phase. The disappearance of PHE intact drug spot at R_f_ 0.12 was accompanied by the appearance of two spots of degradation products at R_f_ 0.45 and 0.55, while the IBU degradation solution showed only the intact drug spot. These results are in agreement with those which concluded that IBU is susceptible only to strong oxidation conditions [23] and that oxidative degradation of PHE resulted in two degradation products [20], Deg1 and Deg2 (Fig. 2). Absence of IBU degradation was confirmed by extraction of the drug spot twice with methanol and injecting 20 µL aliquots of the resulting solution in triplicates into the column. The process was completed as described under “Preparation and structure elucidation of PHE degradation products”; a single peak for IBU was obtained. On the other hand, the two degradation products of PHE (Deg1 & Deg2) were separated via preparative TLC, purified and identified using IR (Fig. 3) and mass spectrometry (Fig. 4).

The IR spectra of PHE degradation products (Fig. 3) show appearance of aromatic ketone bands around 1640 cm^−1^ and oxide band in Deg2 spectrum at 1453 cm^−1^_._ Mass spectra of Deg1 and Deg2 (Fig. 4) showed peaks at m/z 184 and 195 consistent with the molecular weight of their proposed structures. The suggested pathway for PHE degradation is shown in Fig. 2.

### Method development and optimization

**Fig. 5:**
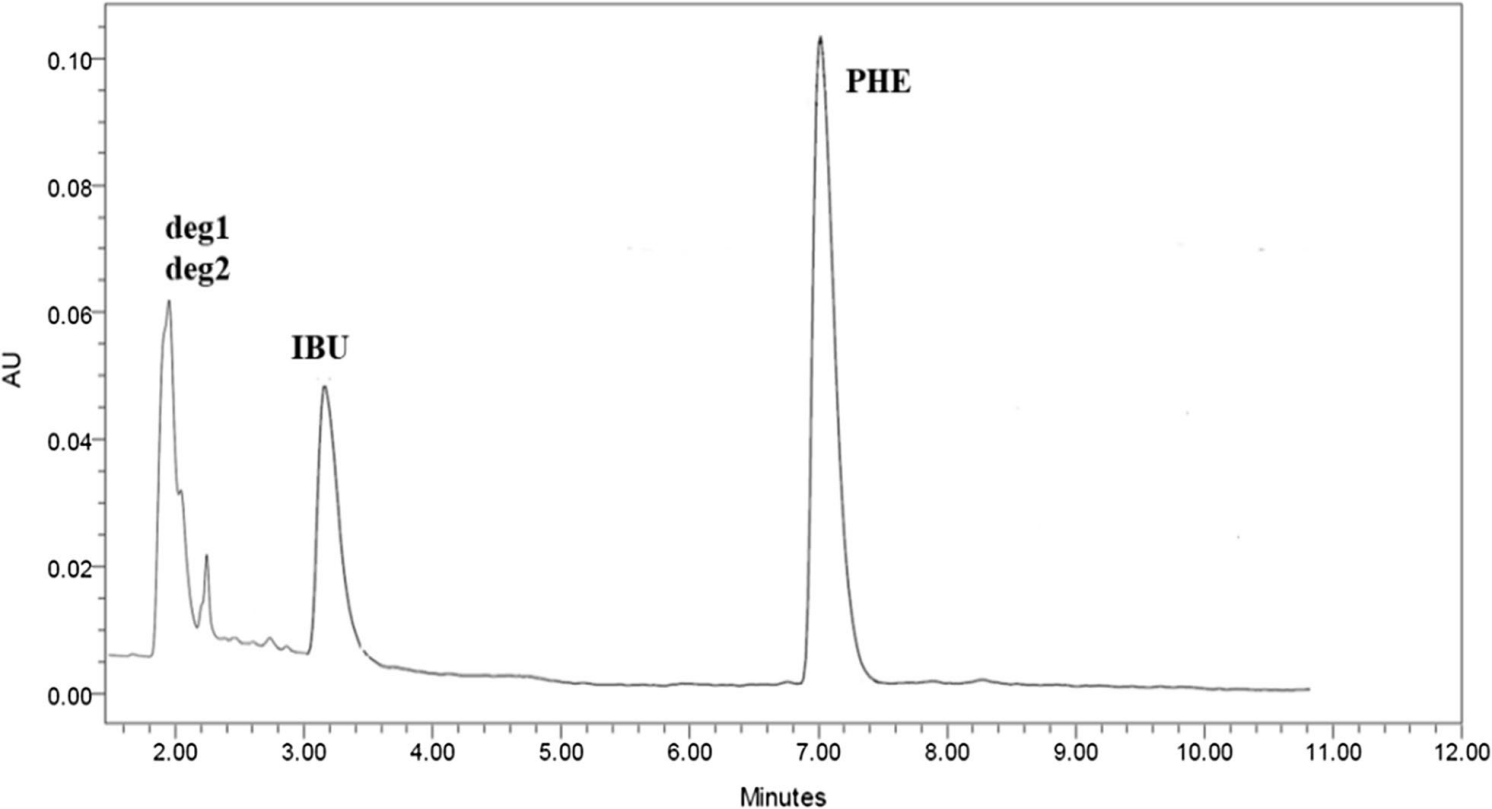
HPLC chromatogram of Deg1, Deg2, IBU and PHE (10 μg/mL each)

The main goal of this study was to provide a sensitive, accurate and selective HPLC method for assaying IBU, PHE and its oxidative degradants simultaneously in their quaternary mixture and in dosage forms. Important factors affecting the efficiency of the HPLC technique were studied carefully. Levels of a given factor were changed while the other chromatographic conditions remained unchanged. Many trials have been done on several types of columns to achieve optimum separation, e.g., XTerra MS (100 × 4.6 mm, 5 µm), C18 X-bridge shield RP and YMC-C8 column (100 × 4.6 mm, 3 µm). The C18 columns showed very bad retention and the peaks were highly overlapped, while C8 showed best retention and resolution. Different elution modes and mobile phases with various compositions and ratios have been tested for ideal separation. By using isocratic elution and a mobile phase of 0.1% hexane sulfonic acid sodium salt monohydrate and methanol (80:20% v/v), IBU could not be separated. We used also buffer: methanol in different ratios (70:30 and 30:70 v/v), but the peaks were severely overlapped. All solvent compositions and ratios failed to separate the four compounds** (**IBU, PHE, Deg1& Deg2) using isocratic elution; thus, gradient elution was utilized. Again, several solvent combinations of methanol or acetonitrile with buffer or hexanesulfonic acid were used in gradient mode. Finally, YMC-C8 column was used and gradient elution using 0.1% hexane sulfonic acid sodium salt monohydrate and acetonitrile as solvents. The optimum gradient started with hexane sulfonic acid sodium salt monohydrate and acetonitrile at ratio 80:20 v/v, and after 3 min the ratio changed into 60:40 v/v till the end of the run. Various flow rates 0.8, 0.9, 1 and 1.2 mL/min were tested and 1 mL/min was the best regarding separation and resolution. The best UV wavelength used for detection was 220 nm regarding the sensitivity of the two drugs. Different temperatures were applied to investigate best column temperature for separation and 35 °C was the optimum. Good chromatographic separation was achieved with tR of 2.0, 2.2, 3.2 and 7.0 min for Deg1, Deg2, IBU and PHE, respectively (Fig. 5). The system suitability parameters were calculated for the method where acceptable values were obtained within the recommended ranges as shown in Table 1.

**Table 1:** System suitability parameters of the proposed HPLC method

Parameter	IBU	PHE	Reference values [47]
Retention time (t_R_) [min]	3.2	7.0	NA
Resolution (R_s_)^a^	7.86		R_s_ > 1.5
Selectivity factor (α)^a^	1.71		α > 1
Tailing factor (T)	0.97	1.71	T < 2
Capacity factor (K’)	2.73	5.30	1 < K’ < 10
Column efficiency (N)	2196.32	2080.36	N > 2000
Height equivalent to theoretical Plate (HETP) [mm]	0.0198	0.0455	NA

^a^Resolution and selectivity factors are determined between the peaks of IBU and PHE

### Molecular dynamic simulation

Molecular simulation methods have been applied to the modeling of reversed-phase liquid chromatography. The purpose of these simulations was to provide a molecular-level understanding of the structure and dynamics of the bonded phase and its interface with the mobile phase, the interactions of analytes with the bonded phase, and the retention mechanism for different analytes [56].

In RP-HPLC, solutes are eluted in order of decreasing polarity where retention increases with decreasing polarity. Prediction of retention time has become valuable, powerful, and routine in chromatographic method development. Depending on the experimental design, researchers may be interested in retention data, or may use them to infer additional information [41, 57].

The developed method was applied to analysis of two mixtures under varying chromatographic conditions. The experimental retention times for both IBU, PHE and IBU degradation products are summarized in Table 1.

**Fig. 6:**
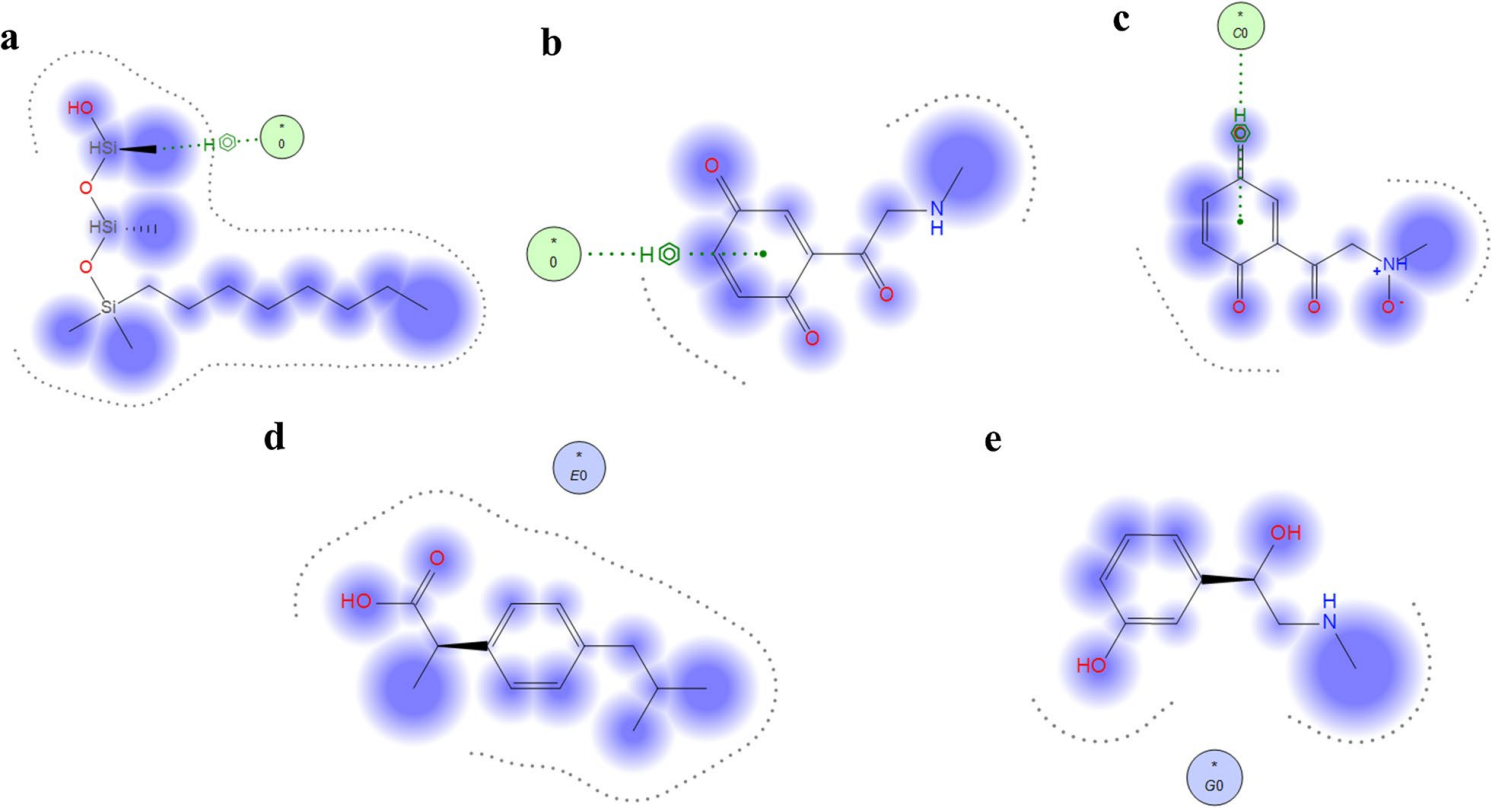
Diagram showing: **a** the composition of YMC-C8 column; **b** 2D interaction of Deg1 and the reversed phase stationary phase Si–O showing arene-H interaction; **c** 2D interaction of Deg2 and the reversed phase stationary phase Si–O showing arene-H interaction; **d** 2D interaction of IBU and the reversed phase stationary phase Si–OH; **e** 2D interaction of PHE and the reversed phase stationary phase Si–OH

**Fig. 7:**
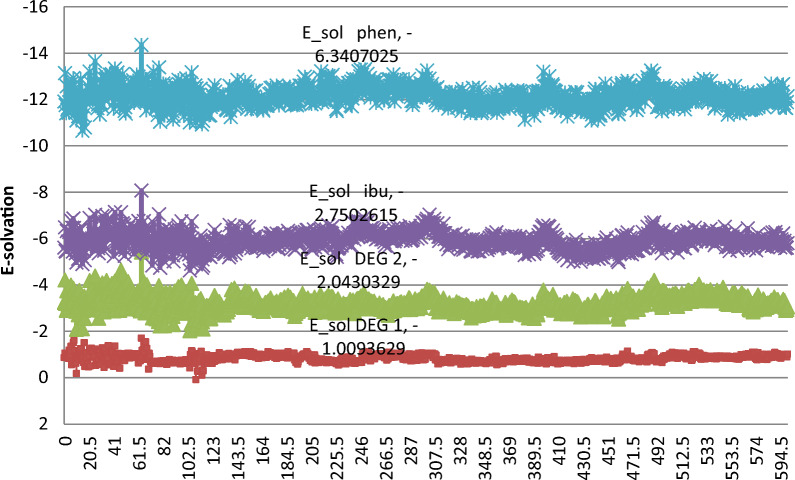
A calibration plot for each component showing the simulation time versus its solvation energy

Before conducting the simulation, dielectric constant (DEC) was adjusted first at 21.58 and then adjusted at 25.56 as the proposed method is gradient. DEC is equivalent to the total ratios of the mobile phase, as we cannot introduce the ratios of the solvent as it will give inaccurate simulation results. Here, the composition of the column plays a serious role in the interaction of the drugs with the stationary phase. The Reversed-phase C_8_ had only an octa chain which could provide hydrophobic interactions; however, using YMC-C8, which offered the same interaction as reversed-phase C_8_, beside it will provide hydrogen bond donor owing to the embedded (Si–OH) group (Fig. 6a) in the bonded phase ligand. Thus, in this work, we used an YMC-C8 column where the interactions between the drugs and the stationary phase were conducted, and the binding energies were calculated. In the case of oxidative degradation of PHE, the resulting degradants and the intact drug were simulated under the mentioned conditions, and after a thorough examination of the resulting trajectories, we found that both degradants and the intact drug interact with the stationary phase in several different modes. The interactions between drugs and the column depend on polarity as the least polar drug will be the most retained with stationary phase and the most polar drug will be the least retained with stationary phase. For Deg 2, there is positive and negative charge on N-oxide group which increase polarity of the component, and also quinone group make tautomerism into its corresponding phenolic groups and that make this component the most polar between four components, so it elutes first with binding energy of -1.18 kcal/mol, as seen in Fig. 6b. Deg 1 shows less polarity as there is no positive and negative charge, so it elutes second with binding energy of -3.16 kcal/mol as shown in Fig. 6c. The intact drug molecules were found to form hydrophobic van der Waals interactions. For IBU, there is free carboxylic group despite methyl group which make weak hydrophobic van der Waals interaction with stationary phase, so it elutes third with binding energy of -3.35 kcal/mol as shown in Fig. 6d. For PHE, it shows strong van der Waals interaction with stationary phase due to phenyl group which make the drug the highest retained component with binding energy of -6.67 kcal/mol as shown in Fig. 6e. Due to the diversity of the interactions between the analyzed components and the stationary phase, these observations were not enough to prove the arrangement of the separated components regarding their binding to the stationary phase. So, after conducting the molecular dynamics simulation, the solvation energies were calculated from the emerging trajectories. A calibration plot for each component was generated by plotting the simulation time versus its solvation energy. Figure 7 arranges the oxidative degradations and the intact drugs according to their solvation energies throughout the whole simulation time. The data extracted from the calculated solvation energies were in line with the binding energy data and experimental data from the proposed chromatographic method. To the best of our knowledge, this is the first time molecular docking was used in chromatographic approaches to assess the interaction of the analytes with the stationary phase and the prediction of the elution order, and to confirm the outcomes of the proposed method by revealing which drug will be more and which one will be less retained in the stationary phase.

### Assessment of the greenness of the developed method

The greenness profile of the proposed method was assessed and ranked according to Analytical Eco Scale tool (AES tool) as a well-established method and was reported to provide reliable and precise results about method greenness [34, 40, 47–53].

The greenness profiles of the developed method and a reported method [17] using AES tool were compared and the results obtained are presented in Table 2. The scores calculated for the newly developed reversed phase HPLC method and the reported method were 88 and 84, respectively. Both methods are green having a score above 50; an AES score of more than 75 is considered excellent for eco-friendly analysis. Although both methods have equal waste and occupational hazards scores, the proposed method has the advantage of using less and greener organic solvents compared to the reported method.

**Table 2:** Comparison of the greenness profiles of proposed and a reported method using ESA tool

Reagents and instruments	Proposed method	Reported method [17]
Chloroform	0	2
Methanol	0	6
Ammonia	0	2
0.1%hexanesulfonic acid	0	0
Acetonitrile	6	0
Waste	3	3
Occupational hazards	3	3
Total penalty points	12	16
Total score	88	84

### Method validation

Validation of the proposed technique was carried out in accordance with the ICH guidelines [58].

#### Linearity and range

The linearity for each drug was estimated by investigating various concentrations of IBU (10–100 µg/mL) and PHE (0.3–10 µg/mL). The regression equations result and their corresponding relative standard errors accompanied by the LOD and LOQ were shown in Table 3.

**Table 3:** Assay parameters and validation data for determination of ibuprofen and phenylephrine by the proposed HPLC method

	PHE	IBU
Range (µg/mL)	0.3–10	10–100
Slope	989,994	1611.40
Intercept	528,735	− 11,978
SE of the slope	14,376.11	18.4132
SE of the intercept	68,432.38	1143.568
Correlation coefficient (r)	0.9995	0.9997
LOD^a^ (µg/mL)	0.4399	2.75
LOQ^a^ (µg/mL)	0.30	8.33
Accuracy^b^	99.44%	99.45%
Repeatability^c^	1.34	1.09
Intermediate precision^d^	1.47	1.23
Specificity (mean ± SD)^e^	99.67 ± 1.67	100.75 ± 1.44
Robustness	1.94	1.19
Flow rate 1.0 + 0.1 mL/min		
Mobile phase; 80:20, 60:40		
80.8:20.2, 60.6:40.4	1.87	1.31

^a^Limits of detection and quantitation are determined via calculations, LOD = 3.3 σ/S (SD of the residuals/slope), LOQ = 10 σ/S (SD of the residuals/slope), where σ is the standard deviation of *y*-intercepts of regression lines and *S* is the slope of the calibration curves

^b^Mean (n = 3) R% of three concentrations within the linearity ranges

^c^Intra-day precision (*n* = 3), three concentrations repeated three times within the same day

^d^Inter-day precision (*n* = 3), three concentrations repeated three times in three successive days

^e^Mean (n = 3) R% of laboratory prepared mixtures containing IBU, PHE and its degradation products

#### Accuracy

Accuracy was assessed by applying the proposed HPLC method on pure samples with various concentrations within their linearity ranges, where satisfactory recoveries were attained (Table 3).

#### Precision

On the same day, three different concentrations of IBU (10, 40, 60 µg/mL) and PHE (0.3, 1.0 and 3.0 µg/mL) were assessed three times for repeatability. On three different days, the previous procedures were performed for the analysis of previously selected concentrations to assess intermediate precision. Relative standard deviations (RSD%) values indicated low deviations and high repeatability (Table 3).

#### Robustness

Robustness of the method was studied by deliberately modifying certain experimental conditions separately, namely the flow rate and the percentage of mobile phase ratio**.** As revealed in Table 3, no significant difference was observed on changing the organic solvent ratio in the mobile phase (20 ± 1.0%) or the flow rate (1.0 ± 0.1 mL/min); reasonable RSD% were obtained.

#### Specificity

Various laboratory-prepared combinations with different proportions of the investigated drugs (IBU and PHE) with the degradants (Deg1 & Deg2) were analyzed. The recovery percentages and SD shown in Tables 3 and 4 confirm that the method can successfully be applied to the estimation of ibuprofen and phenylephrine hydrochloride in combined pharmaceutical dosage form and in the presence of PHE oxidative degradants.

**Table 4:** Determination of ibuprofen and phenylephrine in Grippostad® tablets and application of standard addition technique using the proposed method

	Grippostad® tablets	Standard addition technique		
	Mean^*^ ± SD	Taken (µg/mL)	Added (µg/mL)	Recovery%*
PHE	99.44 ± 1.38	0.5	0.25	99.20
			0.5	101.15
			1	98.90
		Mean^a^ ± SD		99.75 ± 1.22
IBU	99.78 ± 1.27	20	10	99.80
			20	99.82
			40	101.34
		Mean^a^ ± SD		100.32 ± 0.88

^*^Average of three determinations

### Application the proposed method to Grippostad® tablets and statistical comparison

**Fig. 8:**
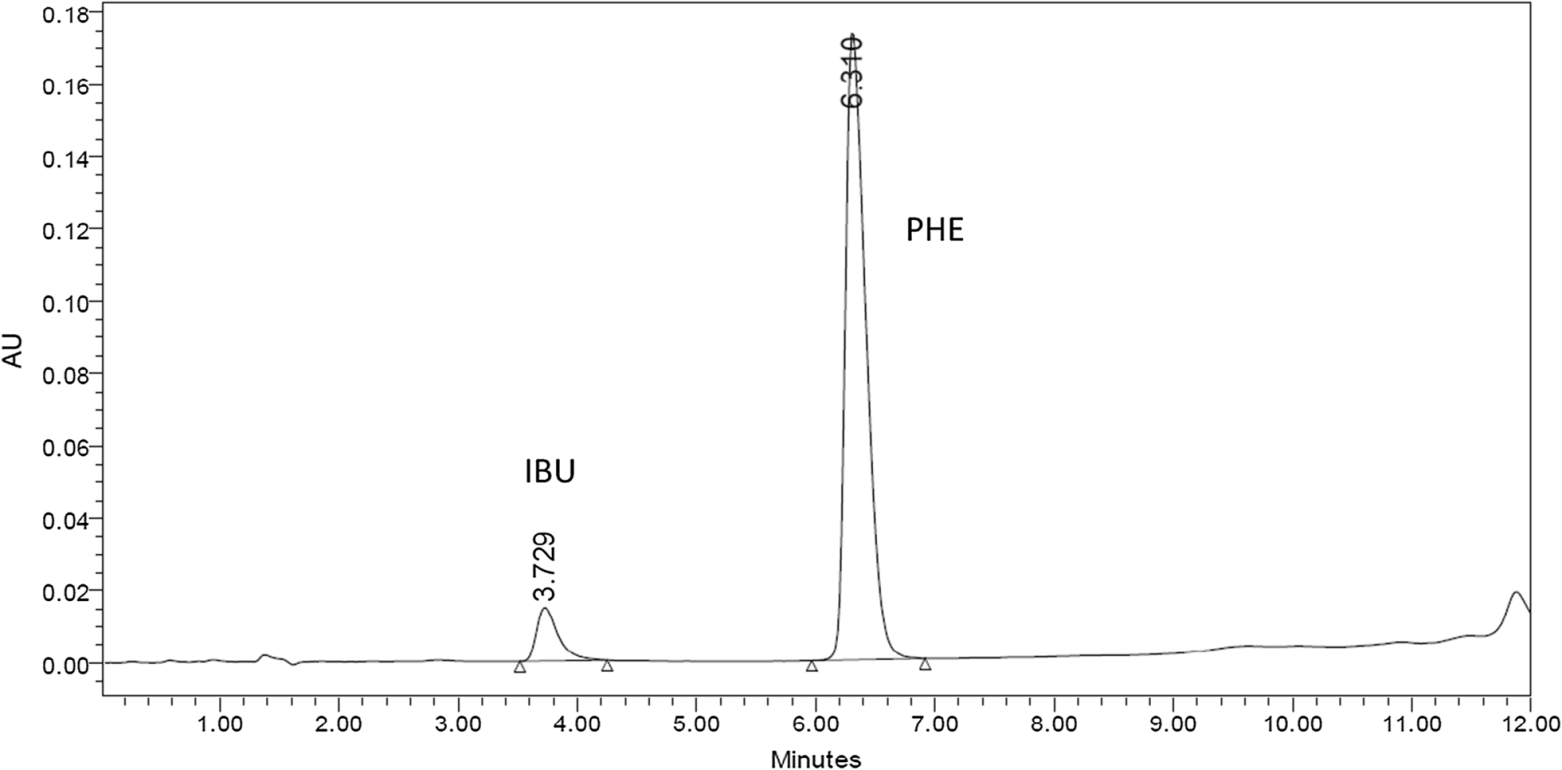
HPLC chromatogram of IBU and PHE combination extracted from Grippostad® tablets

The developed method was successfully applied for assaying IBU and PHE in Grippostad® tablets. As the powder was sonicated, filtered and rinsed several times with methanol, there was no interfering peaks from the excipients and good separation was achieved as shown in Fig. 8.

On applying the standard addition technique, excellent recoveries IBU and PHE, Deg1 and Deg2 were obtained with high precision and accuracy as shown in Table 4.

The results obtained from IBU and PHE analysis in pure powder and the official method [1] of analysis were compared statistically. T and F tests confirmed no significant difference between the developed method and the official one as shown in Table 5.

**Table 5:** Statistical comparison for the results obtained by the proposed HPLC method and the official method [1]

Parameter	Proposed method		Official method [1]	
	PHE	IBU	PHE	IBU
Mean of recoveries^a^	99.44	99.78	100.02	99.57
SD	1.38	1.27	0.85	1.09
Variance	1.92	0.13	0.73	1.19
N	5	5	5	5
Student’s t-test (2.30)^b^	0.78	0.24	–	
F-test (6.38)^b^	2.60	0.11	–	

^a^Average of three determinations

^b^Values between parenthesis are corresponding to the theoretical values of *t* and *F* (*P* = 0.05)

## Conclusion

An eco-friendly HPLC method has been developed for the determination of IBU, PHE and An eco-friendly HPLC method has been developed for the simultaneous determination of IBU, PHE and its oxidative degradants. The proposed approach is simple, accurate and precise and can be utilized in the analysis of binary mixture of IBU and PHEN in presence of the oxidative degradants in pure form and in dosage form. The method has been proved to be green with respect to AES metrics. The method can monitor PHE degradation which is proved to be a risk factor on patients’ health. MDS was used to analyze the elution behavior of the analytes and its results was comparable to the method’s results. AES metric was used for calculating of the greenness of this method and comparison to a published method.

## Data Availability

All data generated or analyzed during this study are included in this published article.
